# Enzymology, Histological and Ultrastructural Effects of Ar-Turmerone on *Culex pipiens pallens* Larvae

**DOI:** 10.3390/insects11060336

**Published:** 2020-05-30

**Authors:** Jia Liu, Diana Fernandez, Yanjin Gao, Pierre Silvie, Yongdong Gao, Guanghui Dai

**Affiliations:** 1 Laboratory of Plant Health and Natural Products, School of Agriculture and Biology, Shanghai Jiao Tong University, Shanghai 200240, China; iam_liujia@sjtu.edu.cn (J.L.); gao301301@sjtu.edu.cn (Y.G.); 2 IRD, Cirad, University of Montpellier, IPME, 911, Avenue Agropolis, CEDEX 5, 34394 Montpellier, France; diana.fernandez@ird.fr; 3 IRD, UMR IPME, 34AA001 Montpellier, France; pierre.silvie@cirad.fr; 4 AIDA, University of Montpellier, CIRAD, CEDEX, BP 34398 Montpellier, France; 5 Shanghai Agriculture Extension and Service Center, Shanghai 201103, China; gaoyongdong82@163.com

**Keywords:** phytochemical, mosquito, immature stages, mechanism

## Abstract

Our previous article demonstrated that ar-turmerone ((6S)-2-methyl-6-(4-methylphenyl)-2-hepten-4-one) extracted from *Curcuma longa* L. has a significant larvicidal activity against the fourth instar larvae of *Culex pipiens pallens*. To reveal the effects of ar-turmerone on C. pipiens pallens larvae, light microscopy and transmission electron microscopy were used to observe the histological and ultrastructure changes in muscle and digestive tissues of fourth instar larvae. It was also revealed by detecting the activity of the acetylcholinesterase (AChE) enzyme and three detoxifying enzymes, including carboxylesterase (CarE), glutathione-S-transferase (GST) and Cytochrome P450 monooxidases (P450). The observation under the light microscope showed that the larvae displayed a disruption of myofibril in ventral muscle cells, the disappearance of nucleolus in the malpighian tubule cells, and the exfoliation of the brush border in midgut epithelial cells, 24 h after treatment. The observation under the transmission electron microscope displayed disorganized Z-lines in the ventral muscle cells, and dissolved membrane of mitochondria, nuclear and endoplasmic reticulum in abdominal cells. The enzymatic activity results showed that ar-turmerone significantly increased the level of detoxifying enzymes, while the activity of AChE was not obviously affected. All the results suggest that the larvicidal mechanism of ar-turmerone is estimated to be stomach poison and the active sites might be the muscle and digestive tissues, and the mode of action of ar-turmerone may be unrelated to AChE.

## 1. Introduction

Mosquitoes are transmission vectors of protozoans, filariae or arboviruses, causing diseases that seriously imperil human health, including malaria, yellow-fever, dengue, chikungunya and Zika fevers [1]. *Culex pipiens pallens* is the primary vector of bancroftian filariasis and epidemic encephalitis, and is also a potential vector of West Nile Virus (WNV) in China [2]. To control the diseases spread by mosquitos, source reduction is a straightforward way to solve the problem, which includes elimination of larval habitats or applying larvicides to impair mosquito development. Although chemical larvicides are the main choice, for their rapid action and high efficacy, their long- term abuse has given rise to environmental concerns [3]. Repeated applications of synthetic pesticides may cause the rapid development of insect resistance [2,4,5]. In addition, with only a few mosquito larvicides (Temephos, *Bacillus thuringiensis* subsp. *israelensis*) existing on the market, it is urgent to develop novel eco-friendly pesticides for larval management.

Botanical-based insecticides are considered effective alternatives to chemical substances for mosquito management, as they are safe, biodegradable and ecologically acceptable [6,7]. More importantly, botanical pesticides may display a new mode of action [8,9,10]. For example, nicotine can affect acetylcholine receptors in the nervous system, hydrolysable tannins can negatively influence insect growth, and monoterpene esters can cause disturbances in the nervous system, leading to paralysis and mortality [11]. In China, *Curcuma longa* L. (Zingiberaceae) is a traditional herb which has been reported to have antimicrobial [12,13], mosquitocidal [14,15] and pharmaceutical [16] activities. In a previous study, ar-turmerone, a sesquiterpenoid (C_15_H_20_O) extracted from *C. longa*, has demonstrated significant larvicidal activity against the fourth instar larvae of *Culex pipiens pallens*, with a LC_50_ value of 138.86 ppm [17]. This LC_50_ value is higher than that for organophosphate insecticide Temephos, which is commonly recommended by WHO for the larvae control of a range of mosquito species. However, a number of reports indicate the emergence of mosquito resistance to Temephos [18,19]. In consideration of the deficiency of mosquito larvicides and the potential of ar-turmerone, more research on the effects of ar-turmerone against mosquito larvae is needed.

Therefore, we firstly explored the effects of ar-turmerone on the digestive and abdominal muscle tissues of the larvae, using histological and ultrastructural methods. To better identify the mode of action of the molecule, we further analyzed the main enzymes known to be involved in different mechanisms. Acetylcholinesterase (AChE) is an important target-related enzyme for many pesticides in the nervous system. Its inhibition is provoked by essential oils used against the larvae of mosquitoes. The detoxifying enzymes Carboxylesterase (CarE), Glutathione-S-transferase (GST) and Cytochrome P450 (P450) were analyzed too, because they become involved after the insecticides enter the insects [20,21].

## 2. Materials and Methods

### 2.1. Mosquitoes

The larvae of *C. pipiens pallens* were collected from Shanghai Research and Development Center for Pesticides (3111′ N; 12115′ E, 3.84 m). The larvae were kept in open trays at 28 ± 2 °C, under 80.0% ± 5% relative humidity (RH) and under 12 h light and 12 h dark photoperiods. A yeast suspension (5%) (mixing 5 g yeast powder into 100 mL water) was used as the food source. Once the larvae had transformed into the pupal stage, the pupae were collected and transferred from the culture trays to plastic circular containers filled with 500 mL of water. These containers were kept in a 35 × 35 × 35 cm mosquito cage for adult emergence. After providing a 10% sugar solution, the adult female mosquitoes were allowed to feed on the blood of mice. Subsequently, new plastic circular containers filled with 500 mL of water were placed in the cage as oviposition substrates.

### 2.2. Ar-Turmerone

Ar-turmerone (95.0% purity) was isolated and purified from the rhizome extract of *C. longa* according to the method described by Liu et al. (2018) in the laboratory of plant health and natural products [17], Shanghai, Jiaotong University.

### 2.3. Treatment of Larvae, Pupae and Eggs by Ar-Turmerone Solution

The larvicidal bioassay was performed using the method described by Liu et al. (2018) [17]. Pupal mortality was tested by the method of Chellasamy et al. (2012) [22] and ovicidal experiments were conducted according to the method of Govindarajan et al. (2011) [23]. Ar-turmerone was diluted to different concentrations (10,000, 12,000, 14,000, 16,000, 18,000 ppm) with 1% DMSO (Dimethyl sulfoxide) - water. The solution of each concentration was transferred into 6-cell culture plates. A total of 15 to 20 first to third instar larvae or pupae were placed in the cell culture plates respectively. Larvicidal or pupicidal activities were evaluated after 24 h of exposure in the ar-turmerone solution, and larvae or pupae that showed no response to a gentle prodding with a fine needle were recorded as dead. For ovicidal experiment, 30–50 eggs were pre-exposed to each concentration of ar-turmerone for 24 h, then transferred to distilled water. The hatching assessment was conducted 48 h later with microscope (Olympus, SZ61, Japan) observation. The data were assessed for the ability to inhibit egg viability, using the following formula:

$$unhatchability=\frac{Number\;of\;unhatched\;larvae}{Number\;of\;tatol\;eggs}\times 100\;\left(\%\right)$$

1% DMSO water solution was used as the negative control, Temephos (1% sand granule) (D BASF, Shanghai lican trading co., LTD) as the positive control. All treatments and controls were replicated three times.

### 2.4. Treatment of Fourth Instar Larvae by Ar-Turmerone Solution for Histological Study

50–100 fourth instar larvae were treated with ar-turmerone dissolved in 1% DMSO water solution (200 ppm) (LC_80_ dosage) according to the method described by Ma et al. (2017) [24]. Larvae were taken out of the ar-turmerone solution by tweezers 0, 6, 12 and 24 h after the treatment for further light microscopy observation, and 0 and 24 h after treatment for transmission electron microscopy (TEM) observation. Samples at 0 h were used as negative control.

### 2.5. Histological Observation

The treated fourth instar larvae were fixed in 4% paraformaldehyde for 24 h. The fixed samples were prepared according to the standard method [25]. The prepared samples were cut using the rotary microtome (Leica RM2126, Wetzlar, Germany), stained with hematoxylin-eosin and observed by a light microscope (Olympus, BX51, Tokyo, Japan). The ventral muscle tissues, Malpighian tubules and midgut digestive cells of treated larvae were observed and compared to control.

### 2.6. Transmission Electron Microscopy (TEM)

The sample processing was performed according to Ma et al. (2017) with a little modification of the fixed liquid [24]. The phases of the process are as follows: the abdominal tissues of the treated fourth instar larvae were pre-fixed in 2.5% glutaraldehyde at 4 °C for 6 h, and then fixed in 1% osmium tetroxide for 2 h and rinsed in 0.1 M phosphate buffer (pH 7.2). Then, the abdominal tissues were dehydrated in an acetone series in ascending order (30%, 50%, 70%, 80%, 90% and 100%, v/v) for 15 min each and in absolute acetone twice for 20 min, and embedded in epoxy resin (Beijing daji keyi technology co. Ltd., Beijing, China). The prepared samples were cut using an ultramicrotome (Leica UC6, Wetzlar, Germany) and observed with a transmission electron microscope (Tecnai G2 spirit Biotwin, Hillsboro, WA, USA).

### 2.7. Enzymological Studies

#### 2.7.1. Preparation of the Samples 

*C. pipiens pallens* fourth instar larvae were treated with 100 ppm (LC_30_ dosage) of ar-turmerone in 1% DMSO water solution. The treatment dosage was referred to Liu et al. (2018) [26]. The samples were collected at 1, 6, 12, 18 and 24 h after treatment, respectively. The larvae treated with 1% DMSO water solution were used as the negative control. The protein concentration was measured by Coomassie blue G-250 (Nanjing Jiancheng Bioengineering Institute, Nanjing, Jiangsu).

#### 2.7.2. Determination of AChE Activity

AChE activity was determined in accordance with the method of Chen et al. (2018) [27]. A spectrophotometric assay was used in a multi-detection microplate reader (Infinite M200 PRO, Tecan, Switzerland) at 412 nm. The activity was assessed in vivo, and the reaction mixture included 25 uL of enzyme extract, 50 uL of 0.4 mM 5,5′-Dithiobis (2-nitrobenzoic acid) (DTNB) (Sigma-Aldrich, Shanghai, China), 50 uL of 0.4 mM acetylthiocholine iodide (ATChI) (Sigma-Aldrich, Shanghai, China) and 50 uL of sodium phosphate buffer (0.05 M, pH 7.4) (Sinopharm Chemical Reagent Co., Ltd., Shanghai, China). The reading was taken after incubation for 10 min at 37 °C. Each treatment was replicated three times.

#### 2.7.3. Determination of GST, CarE and P450 Activities

GST activity was determined by the GST assay kit (A004) (Nanjing Jiancheng Bioengineering Institute) for the in vivo assay. GST catalyzes the combination of GSH (glutathione) and 1-Chloro-2,4-dinitrobenzene (CDNB) to produce glutathione dinitrobenzene complex and hydrochloric acid. A spectrophotometric assay was used in a multi-detection microplate reader (Infinite M200 PRO, Tecan, Männedorf, Switzerland) at 412 nm. CarE activity was tested by the CarE assay kit (A133) (Nanjing Jiancheng Bioengineering Institute) for the in vivo assay. CarE catalyzes the formation of naphthalene ester from acetic acid-1-naphthalene ester, with a spectrophotometric assay at 450 nm. P450 activity was determined by the P450 Elisa assay kit (Shanghai Chunshi Biotechnology Co., Ltd., shanghai, China) for the in vivo assay and a spectrophotometric assay was used in a multi-detection microplate reader at 450 nm.

### 2.8. Data Analysis

All the data were analyzed by SPSS 17.0 statistical software (SPSS Inc., Chicago, IL, USA). All the data in the tables are from three replicates and described as mean ± SE. All the data in the figures are also from three replicates and expressed as the mean ± SD. The results of all the enzyme assays were analyzed by one-way ANOVA, followed by Duncan test, at *p* < 0.05.

## 3. Results

### 3.1. Pupicidal and Ovicidal Activities of Ar-Turmerone against C. pipiens Pallens

According to the results shown in Table 1, the pupicidal and ovicidal activity of ar-turmerone were concentration-dependent. The LC_50_ values were 13,172.03 and 11,040.19 ppm, respectively. The result was comparable to the positive control 1% Temephos, which generated a 90% pupae mortality at 20,000 ppm, and caused no egg mortality at 40,000 ppm.

### 3.2. Larvicidal Activities of Ar-Turmerone against the First to Third Instar Larvae

On the basis of confirming the specificity to fourth instar larvae, ar-turmerone was further tested for its activities against different larvae stages, from first to third. The LC50 values were 27.17, 59.66 and 85.71 ppm for first to third instar larvae 24 h after treatment, respectively (Table 2), which indicated that the larvicidal activity of ar-turmerone decreased as age increased.

### 3.3. Toxicity Behavior Observations of Larvae Treated by Ar-Turmerone

The fourth instar larvae treated with ar-turmerone solution (200 ppm) showed a series of toxicity behaviors. The larvae exhibited excitation during 1 h of treatment, and then began convulsion, which would gradually affect the normal movement 6 h after treatment, and then became quiescent 12 h after treatment. They were finally paralyzed to death 24 h after treatment. White flocs were observed in the excretory duct of the tail. Contrarily, the control larvae were excited for the first few minutes, but were back to normal soon.

### 3.4. Histological Observations

**Figure 1:**
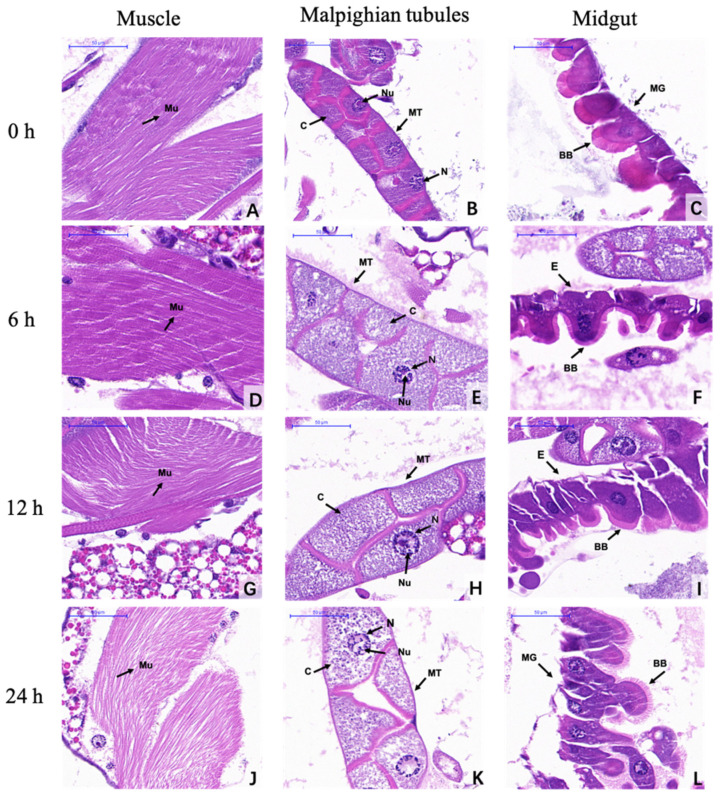
Microstructural changes of *C. pipiens pallens* fourth instar larvae after treatment with ar-turmerone (200 ppm). (**A**–**C**) The muscle fibers showed regular and dense pattern of staining (**A**). The cytoplasm was full, the nucleus and nucleolus were kept integral in the cells of the Malpighian tubule (**B**), and the brush border in the epithelial cells of the midgut were all completely tight and intact in the control larvae (**C**) (0 h). (**A**–**C**) Bar, 50 µm. (**D**–**F**) The muscle fibers and the brush border in the epithelial cells of the midgut showed no obvious change, while the Malpighian tubule enlarged with cytoplasm thinning after 6 h treatment (6 h). (**D**–**F**) Bar, 50 µm. (**G**–**I**) The muscle fibers were observed to be disordered. Malpighian tubule enlarged with disappearing nucleolus. The epithelial cells in the midgut had protruded with the brush border tending to thin out after 12 h treatment (12 h). (**G**–**I**) Bar, 50 µm. (**J**–**L**) The muscle fibers appeared disordered and sparse. Malpighian tubule enlarged, with understained cytoplasm, abnormal chromatin and disappeared nucleolus. The epithelial cells in the midgut had protruded, with the brush border completely disordered and thinning out after 24 h treatment (24 h). (**J**–**L**) Bar, 50 µm. Mu, Muscle fibers; C, Cytoplasm; MT, Malpighian tubules; MG, Midgut; N, Nucleus; Nu, Nucleolus; BB, Brush border; E, epithelial cells.

The observations under the light microscope showed that ar-turmerone treatment at 200 ppm affected the ventral muscle fibers, Malpighian tubules and midgut cells of fourth instar larvae (Figure 1). Before ar-turmerone treatment (control), the muscle fibers showed regular and dense patterns of staining by hematoxylin-eosin (Figure 1A), the cytoplasm was full, the nucleus and nucleolus of cells in Malpighian tubule were complete (Figure 1B), and the brush border in the epithelial cells of the midgut were all completely tight and intact (Figure 1C). 6 h after treatment, the muscle fibers (Figure 1D) and the brush border the in epithelial cells of the midgut (Figure 1F) showed no obvious change, while the Malpighian tubules enlarged with cytoplasm thinning (Figure 1E). 12 h after treatment, the muscle fibers were observed to be disordered (Figure 1G), and Malpighian tubules enlarged with disappearing nucleolus (Figure 1H). The epithelial cells in the midgut had protruded, with the brush border tending to thin out (Figure 1I). 24 h after treatment, the muscle fibers appeared disordered and sparse (Figure 1J). Meanwhile, the Malpighian tubules were enlarged, with understained cytoplasm, abnormal chromatin and a disappeared nucleolus (Figure 1K). The epithelial cells in the midgut tissues protruded, with the brush border completely disordered and thinning out (Figure 1L).

### 3.5. Transmission Electron Microscopy (TEM) Observations

**Figure 2:**
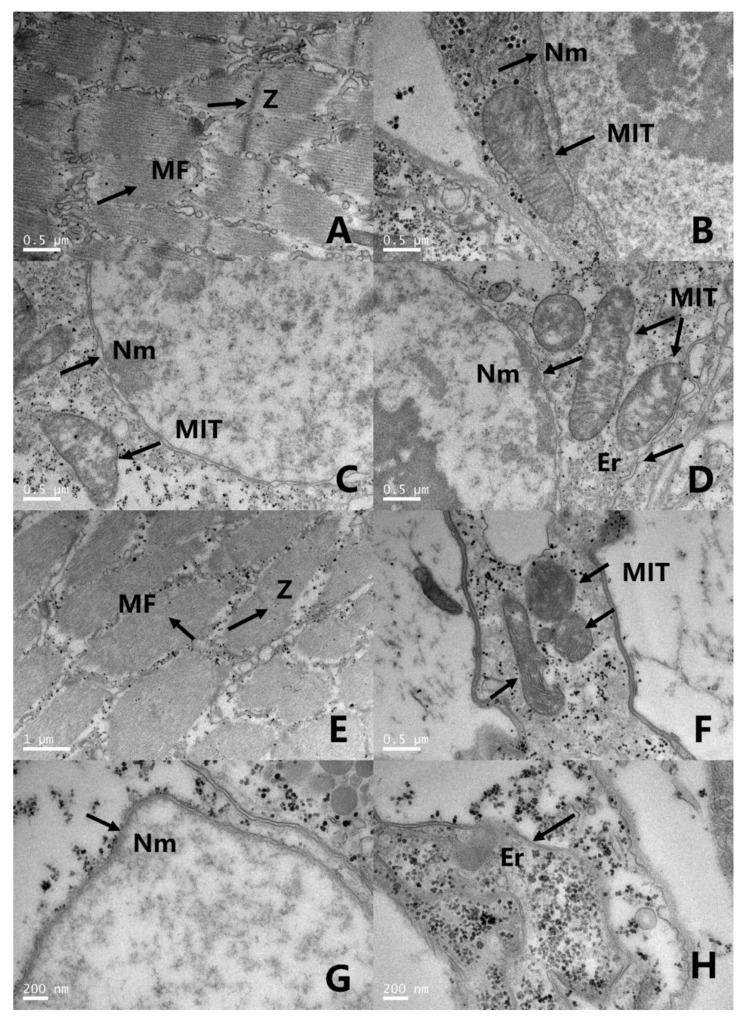
Ultrastructural changes of *C. pipiens pallens* fourth instar larvae after treatment with ar-turmerone (200 ppm). (**A**–**D**) Control larvae: uniform myofibrils and clear Z-lines could be observed. Mitochondria, endoplasmic reticulum and nucleus complete with intact double membrane structure observed. (**A**–**D**) Bar, 0.5 µm. (**E**–**H**) In the deep paralysis stage (pictures taken at 24 h post-treatment) myofibrils in the ventral muscle were disrupted and Z-lines were faint. Mitochondrial membrane was dissolved with cristae vague. Part of the endoplasmic reticulum and nucleus membrane were also observed dissolving. (**E**) Bar, 1 µm. (**F**) Bar, 0.5 µm. (**G**) Bar, 0.2 µm. (**H**) Bar, 0.2 µm. MF, Myofibril; Z, Z-line; MIT, Mitochondria; Nm, Nuclear membrane; Er, Endoplasmic reticulum.

The ultrastructure of the abdominal tissues of fourth instar larvae were observed by TEM (Figure 2). In the control larvae, the ultrastructure observation of the abdominal cells of fourth instar larvae showed uniform myofibrils and clear Z-lines (Figure 2A). The mitochondria, endoplasmic reticulum and nucleus were complete, with intact double membrane structures observed (Figure 2B–D), while 24 h after ar-turmerone treatment, in the deep paralysis stage, the myofibrils in the ventral muscle were disrupted and the Z-lines were faint (Figure 2E), and the mitochondrial membrane was dissolved with cristae becoming vague (Figure 2F). Part of the endoplasmic reticulum and nucleus membrane were dissolved (Figure 2G,H).

### 3.6. Activity of the Enzymes

**Figure 3:**
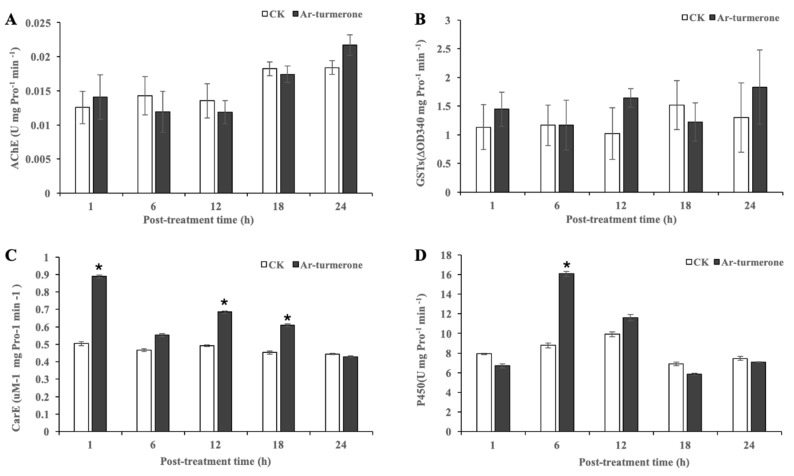
Effects of ar-turmerone on AChE, GST, CarE and P450 activities in *C. pipiens pallens* larvae via in vivo assay. (**A**) AChE in *C. pipiens pallens* larvae. (**B**) GST in *C. pipiens pallens* larvae. (**C**) CarE in *C. pipiens pallens* larvae. (**D**) P450 in *C. pipiens pallens* larvae. Bars represent standard deviations of means. Data in columns with * are statistically different from the control at *p* = 0.05. CK, the control treated by 1% DMSO water solution.

The activities of AChE, GST, CarE and P450 in C. pipiens pallens larvae killed at a dose of 100 ppm ar-turmerone were tested at 1, 6, 12, 18 and 24 h after treatment, respectively (Figure 3). The results showed that the AChE activity was not significantly affected by the treatment (Figure 3A). The activities of GST, CarE and P450 were obviously increased in the treated insects. The GST activity (Figure 3B) increased over 24 h and reached the maximum at 24 h, but the data did not show the statistical difference between the treated and control insects. The CarE activity (Figure 3C) increased to the maximum at 1 h and then decreased to the lowest at 24 h. The overall activity was significantly induced with time, compared to the control, except at 24 h. The ar-turmerone also showed an excellent effect on P450 activity (Figure 3D). P450 activity was inhibited at 1 h, then induced to the maximum at 6 h and reduced to the lowest at 18 h, and finally reduced to the control level at 24 h.

## 4. Discussion

In this study, through the evaluation of the mosquitocidal activities of ar-turmerone against the immature stages of *C. pipiens pallens*, we firstly demonstrated that ar-turmerone is only active against mosquito larvae. This result is similar to the chemical larvicide temephos (1%), which is also active only against mosquito larvae. The larvicidal activity of ar-turmerone decreased with the instar of the larvae, but LC_50_ values for the first to third instar larvae remained below 100 ppm (138 ppm for fourth instar). This result is comparable with the earlier work of Abdul et al. (2000), who reported the larvicidal activity of n-hexadecanoic acid extracted from *Feronia limonia* (Linn), with LC_50_ values of 129.24, 79.58 and 57.23 ppm against the fourth instar larvae of *Culex quinquefasciatus*, *Anopheles stephensi* and *Aedes aegypti*, respectively [28]. This result is similar to that of the natural compound palmitic acid, extracted from *Millettia pinnata* (L.) seeds, which showed LC_50_ values of 34.50, 42.96 and 85.61 ppm against the third instar larvae of *C. pipiens pallens*, *A. aegypti* and *Aedes albopictus*, respectively [29]. Our results also gave lower LC_50_ values than those obtained by Chellasamy et al. (2012) with methanol crude extract of *Artemisia nilagirica* (Clarke). These authors reported LC_50_ values of 272.50, 311.40, 361.51 and 442.51 ppm, respectively, against the first to fourth instar larvae of *A. stephensi*, and 300.84, 338.79, 394.69 and 470.74 ppm, respectively, against the first to fourth instar larvae of *A. aegypti* [22].

As many of the existing pesticides, such as organochlorines and organophosphate, are toxic to humans or other non-target animals, it is necessary to focus on understanding the mode of action of a new insecticidal substance. In our study, the morphological and physiological effects of ar-turmerone were investigated via histological and ultrastructural observations and enzymatic activity. According to the results of histological observation, ar-turmerone-treated larvae were damaged in their muscle and digestive tissues. Their muscle fibers, midgut cells and Malpighian tubules showed histological changes. Our histological findings are similar to the ones reported by Daniel et al. (2014) with niloticin extracted from *Limonia acidissima* L., which seriously damaged the midgut epithelial columnar cells of the larvae of *A. aegypti* [30]. The changes in digestive tissue are almost the same as the ones due to *Bacillus thuringiensis*-affected mosquito larvae and photoactivated insecticide K-01-treated larvae [31]. Plant extracts can also cause similar histopathological changes in mosquitoes. Kala et al. (2019) found that cashew nut (*Anacardium occidentale*) shell liquid can lead to significant damage against the third instar larvae of *Anopheles*, with broken tissues in epithelial cells and the peritrophic membrane, and extensive midgut content leakage [32]. These histopathological changes might therefore be the reasons for insect death.

Further, the TEM observation showed that the endomembrane system in the digestive tissue was disrupted. As reported, the poisoning symptoms caused by ar-turmerone were basically similar to the symptoms of the endotoxin in *Bacillus thuringiensis*. Both of them can damage the midgut cells, destroy the endoplasmic reticulum and cause mitochondrial swelling in insects [33,34]. Besides, the symptoms caused by ar-turmerone were largely similar to those shown by Celangulin V-treated *Mythimna separata* (Walker). Celangulin V, as a stomach poison, acts mainly on the plasma membrane of the midgut and the membrane system of organelles [35]. Celangulin V was thought to bind to the membrane receptors in epithelial cells, as demonstrated for the endotoxin of *Bacillus thuringiensis* [35]. Thus, the destruction of the endomembrane system by ar-turmerone may be caused by the combination of ar-turmerone with some membrane receptors, which may change the information of membrane proteins, and lead to osmotic balance disorder and eventually the collapse of cells. However, further research is still needed to test this hypothesis. Muscle tissue is also very important to insects, and damage to it can even lead to insect death. However, few studies exist on mosquito muscle. In the present work, we found that ar-turmerone could damage the structure of muscle cells in *C. pipiens pallens* larvae, through light and TEM observation. These observations of muscle tissues are similar to those of wilforine-treated *M. separata*. The authors thought the death of *M. separata* was associated with muscle lesions, and they concluded that the action site of wilforine may be located in the muscle tissue [24]. Based on this conclusion, we can also suspect that the action site of ar-turmerone is located in the muscle tissue.

To further understand the mode of action, we studied the neuro-related enzyme AChE and three detoxifying enzymes, as few publications on the effects of insecticide on these enzymes in mosquitoes or larvae can be found. In our present study, ar-turmerone increased the activity of detoxifying enzymes CarE and P450 in vivo over certain time periods, whereas there was no significant difference in the AChE activity between treated and non-treated insects under similar conditions. These results suggest that ar-turmerone could not be an AChE inhibitor. This is very different from previous findings, which indicated that a large number of phytochemicals can inhibit AChE activity [29,36]. Further, the results differ from those for organophosphorus [37] and pyrethroid [38] insecticides, which are the main commercial neurotoxic mosquitocides in China. Organophosphorus affects sodium channels by inhibiting the activity of acetylcholinesterase, and the action mode of pyrethrins is also via activation of the sodium channel [39]. The detoxifying enzymes are normally reported to have an important role in allelochemical metabolism and tolerance, though few cases at the biochemical level have demonstrated these roles [40]. The inhibition of GST, CarE and P450 may decrease the possibility of resistance [41,42]. This study showed that ar-turmerone can induce toxicity to *C. pipiens pallens* larvae, but it also increases the levels of detoxifying enzymes, which indicates the possibility of resistance via its induction. This remains speculative, at least for ar-turmerone in the present work. The results were similar to the research reported by Nutchaya et al. (2014), which demonstrated the potential of thymol and 1,8-cineole in inducing acute toxicity in *Plutella xylostella* larvae, but the compounds also increased the levels of detoxification enzymes [43]. The authors also suspected the possibility of resistance as a result of their induction. This phenomenon can be avoided by using intact oils that contain numerous active compounds. The complex mixtures can prevent the development of resistance [43]. Many natural plant products have been reported for the inhibition of detoxifying enzymes, such as extracts of *Melia*, *Amaranthus*, and *Derris* in *Spodoptera exigua* [44,45], or *Alpinia galanga* in *Bactrocera dorsalis* [46]. The aim of this work is also to provide a theoretical basis for the future mosquito larvicide development of the ar-turmerone-rich extract of *C. longa*.

## 5. Conclusions

In conclusion, ar-turmerone was estimated to work as a stomach poison for the larvae of *C. pipiens pallens*, and induces pathological changes in the muscle, Malpighian tubule and midgut. The site of action of ar-turmerone may be located in the muscle tissue and digestive tissue, but further molecular biology studies should be conducted to confirm this. The increasing level of detoxifying enzymes CarE and P450 induced by ar-turmerone raised the possibility of insect resistance emergence, suggesting that it would be better to use the extract of *C. longa* to develop a new botanical larvicide than ar-turmerone alone.

## Figures and Tables

**Table 1:** Activities of ar-turmerone against different live stages of *C. pipiens pallens*.

Compound	Life Stages	Concentration (ppm)	Mortality ± SE (%)	LC_50_ (95%CL) ^a^ (ppm)
ar-turmerone	Larvae ^b^	-	-	138.86 (127.04–150.93)
	Pupae	10,000	13.96 ± 2.93	13,172.03 (12,575.49–13,773.53)
		12,000	25.97 ± 3.27	
		14,000	60.61 ± 11.61	
		16,000	86.04 ± 2.93	
		18,000	94.41 ± 2.83	
	Eggs	10,000	33.27 ± 4.18	11,040.19 (101,07.68–11,748.17)
		12,000	65.14 ± 2.64	
		14,000	82.41 ± 4.04	
		16,000	79.26 ± 0.74	
		18,000	87.92 ± 3.07	
1% Temephos	Larvae	50	100	-
	Pupae	20,000	90	-
	Eggs	40,000	0	-
1% DMSO	Larvae	-	0	-
	Pupae	-	0	-
	Eggs	-	0	-

^a^ The estimated lethal concentrations (LC_50_) were calculated by probit analysis. CL denotes confidence limit. ^b^ The larvae used in the tests were fourth instar and the LC_50_ result has been published in liu et al. (2018) [17].

**Table 2:** LC_50_ value of different ages of *C. pipiens pallens* larvae treated with ar-turmerone.

Compound	Ages	LC_50_ (95%CL) ^a^ (ppm)	χ^2^	*p*-Value
ar-turmerone	1th	27.168 (10.175–51.84)	4.55	0.72
	2th	59.655 (51.460–66.716)	13.02	0.45
	3th	85.713 (75.476–92.796)	5.76	0.84

^a^ The estimated lethal concentrations (LC_50_) were calculated by probit analysis supplied by SPSS 17.0 (SPSS Inc., Chicago, IL, USA). CL denotes confidence limit.
